# Silymarin-Enriched Extract from Milk Thistle Activates Thermogenesis in a Preclinical Model of High-Fat-Diet-Induced Obesity to Relieve Systemic Meta-Inflammation

**DOI:** 10.3390/nu16234166

**Published:** 2024-11-30

**Authors:** Marina Reguero, Guillermo Reglero, José Carlos Quintela, Ricardo Ramos-Ruiz, Ana Ramírez de Molina, Marta Gómez de Cedrón

**Affiliations:** 1 Molecular Oncology Group, IMDEA Food Institute, CEI UAM + CSIC, E28049 Madrid, Spain; marina.reguero@imdea.org (M.R.); ricardo.ramos@imdea.org (R.R.-R.); ana.ramirez@imdea.org (A.R.d.M.); 2 NATAC BIOTECH, Electronica 7, E28923 Madrid, Spain; jcquintela@natacgroup.com; 3 Production and Characterization of Novel Foods Department, Institute of Food Science Research CIAL, CEI UAM + CSIC, E28049 Madrid, Spain; guillermo.reglero@imdea.org; 4 Cell Metabolism Unit, IMDEA Food Institute, CEI UAM + CSIC, E28049 Madrid, Spain

**Keywords:** energy expenditure, thermogenesis, meta-inflammation, aging, obesity, silymarin

## Abstract

Background: Obesity and aging are associated with the progressive loss of brown adipose tissue (BAT), an increase in visceral white adipose tissue (vWAT), and a reduction in subcutaneous white adipose tissue (sWAT). The progressive expansion of visceral obesity promotes a low grade of systemic chronic inflammation (meta-inflammation), contributing to the onset of comorbidities such as type 2 diabetes mellitus (T2DM), metabolic syndrome, and even cancer. Thus, preserving the thermogenic activity of adipose tissue and improving the metabolic flexibility of sWAT could be an effective strategy to prevent the development of metabolic chronic diseases and promote healthy aging. Precision nutrition has emerged as a complementary approach to control the metabolic alterations associated with unhealthy obesity and aging. In a previous work, we described that a silymarin-enriched extract from milk thistle (Mthistle) increased markers of browning and thermogenesis in vitro in human differentiated adipocytes (SGBS). Objectives/Methods: Therefore, this study aims to evaluate the potential of Mthistle to activate thermogenesis in a preclinical model of high-fat diet (HFD)-induced obesity (DIO). Results: Our results demonstrate that Mthistle increases systemic energy expenditure (EE), preserves body temperature after cold exposure, improves insulin resistance, and reduces inflammatory markers in WAT. Conclusions: Based on these results, silymarin-enriched extract from Mthistle may be proposed as a nutraceutical for the management of metabolic chronic diseases and/or accelerated aging.

## 1. Introduction

Sedentary lifestyle and overfeeding constitute a worldwide health problem as they promote accelerated aging, chronic obesity, and associated comorbidities, including metabolic syndrome, type 2 diabetes mellitus, cardiovascular disease, infections, neurodegenerative diseases, and even cancer [1,2].

Chronic obesity, and more specifically, inflammatory visceral obesity, is characterized by the accumulation of cell-cycle-arrested senescent cells. These senescent cells are metabolically very active with an exacerbated inflammatory profile to recruit pro-resolving immune cells for tissue homeostasis. However, during chronic obesity, the accumulation of damaged inflammatory cells leads to alterations in the metabolic function of specific tissues such as adipose tissues (ATs), muscles, the pancreas, and the liver. Similarly, the aging process is characterized by the progressive accumulation of senescent cells along with the loss of thermogenic adipose tissues—mainly brown (BAT) and subcutaneous white adipose tissues (sWAT)—and the progressive increase in visceral fat (vWAT), which underlies the onset of age-related diseases such as type 2 diabetes, immunometabolic conditions, infections, and cancer. Thus, aging and visceral obesity share common features, including low-grade chronic inflammation, systemic metabolic stress, and immune system dysfunction [3]. In this scenario, lifestyle interventions such as physical exercise, intermittent cold exposure, or fasting-mimicking diets have been proposed as preventive strategies to avoid the loss of thermogenic adipose tissues to relieve meta-inflammation in obesity and aging [4].

Precision nutrition has emerged as a promising discipline to design personalized interventions based on the scientific knowledge of the functional effects of ingredients from the diet or from natural sources (nutrigenomics), while considering the genetic susceptibility (nutrigenetics), lifestyle factors, and the nutritional characteristics of individuals [5,6].

Inducing adaptive thermogenesis and preventing the loss of thermogenic adipose tissues (mainly brown and subcutaneous adipose tissues) is an interesting strategy to alleviate the systemic meta-inflammation associated with obesity and aging [7]. Although there is a genetic susceptibility for the development of metabolic alterations, there are opportunities to alleviate these conditions through diet-derived ingredients, including bioactive natural compounds [8,9].

In a previous work, we screened 20 plant-based extracts, approved by EFSA for human consumption, to evaluate their potential in activating thermogenesis and respiratory oxidative capacity in human-differentiated adipocytes [10]. Among them, silymarin-enriched extract from milk thistle (Mthistle) displayed the highest effects on key metabolic targets associated with mitochondrial uncoupling, mitochondrial biogenesis, fatty acid mobilization, and fatty acid β-oxidation [11]. At the molecular level, Mthistle increased the expression of *UCP1*, *SIRT1*, *CKMT*, *TFAM*, and *PPARγ* genes, which are key regulators of mitochondrial function and the uncoupling process [12]. These effects were corroborated by functional experiments where Mthistle reduced the total neutral lipid content associated with the increase in FAO and oxidative respiratory capacity in differentiated human adipocytes [12].

Herein, we conducted a preclinical study to evaluate the potential preventive and therapeutic effects of Mthistle in alleviating the systemic metabolic stress associated with HFD-induced obesity.

## 2. Materials and Methods

### 2.1. Milk Thistle Extract (Mthistle)

Bioactive compounds from the seeds of milk thistle (*Silybum marianum* L. Gaertn) were extracted using a hydroethanolic solution (70:30 ethanol, *v*/*v*) to obtain an extract with 50% silymarin content, as previously described [10].

Briefly, the extraction process began with cleaning and drying the seeds to remove impurities and reduce moisture content. The seeds were then ground into a fine powder to increase the surface area for solvent interaction. A solvent (20 mL per gram of seed powder) was added and maintained at 50 °C under continuous agitation for 4 h. The liquid phase was separated from the spent seeds and concentrated 20-fold. It was then diluted in water, causing a precipitate that was separated from the aqueous fraction by centrifugation. The recovered fraction was finally dried and milled, resulting in an extract containing 50% silymarin, according to the guidelines from The European Pharmacopoeia Monograph for Milk Thistle Dry Extract, refined and standardized (referenced as 01/2024:2071, European Directorate for the Quality of Medicines & HealthCare-EDQM).

### 2.2. Animal Model

To study the potential of Mthistle in the control of metabolic stress associated with obesity, we designed a preclinical model of high-fat diet (HFD)-induced obesity. No formal a priori power analysis was conducted, due to the exploratory nature of this study. However, the sample size was chosen to balance achieving sufficient statistical power and adhering to the principles of the 3Rs (Replacement, Reduction, and Refinement) in animal research. Thus, 28 male C57BL/6HsdOla mice, 6 weeks of age, were divided into three groups: normal-diet control group (ND) (N = 6); high-fat-diet control group (HFD-control) (N = 10); and high-fat-diet Mthistle group (HFD-Mthistle) (N = 12). The HFD-control and HFD-Mthistle groups were induced to develop high-fat diet (HFD)-induced obesity (DIO) by feeding on an irradiated 45% high-fat diet (HFD, Research Diets, D12451) for 12 weeks.

After 12 weeks on a high-fat diet (HFD), a glucose tolerance test was performed to confirm glucose intolerance in the mice before initiating the intervention with or without Mthistle extract for 12–14 weeks. Mthistle was dissolved in drinking water and administered by gastric gavage three days per week.

To determine the intervention doses, we referred to the European Union herbal monograph on *Silybum marianum* (L.) Gaertn. fructus and its main bioactive component, silymarin, which considers doses up to 600 mg/day of silymarin safe for human consumption (reference EMA/HMPC/294187/2013, 19 September 2018). The silymarin content in milk thistle extract varies depending on the extraction method and conditions, typically ranging from 30% to 65% of the dry extract. Therefore, 800 mg of milk thistle extract typically contains between 240 mg and 520 mg of silymarin.

A pilot experiment with six mice tested three different doses of the extract—400, 800, and 1200 mg—over one week. No apparent toxicity effects were observed, as the mice did not exhibit any changes in behavior, physical appearance, or weight, nor any signs of distress or adverse reactions. Consequently, a final dose of 800 mg of the extract per kg of mice was chosen. Drinking water was administered similarly by gavage to the HFD-control group on the same days.

The main readouts of this study included the analysis of insulin and glucose levels in plasma and the activation of thermogenesis. Glucose and insulin tolerance tests and indirect calorimetries were performed. Additionally, the response to cold exposure stress was evaluated by monitoring rectal temperature. Body weight, food intake, and drinking water were recorded weekly. Food intake was calculated as the total food consumed in each cage, divided by the number of mice in that cage, and multiplied by the number of cages per treatment group.

This study was conducted at the National Cancer Research Center (Centro Nacional de Investigaciones Oncológicas, CNIO) in Madrid, Spain. Animals were housed in standard cages with 12 h light/dark cycles, and environmental enrichment was included to promote natural behaviors. All experimental procedures were approved by the Ethics Committee of CNIO-ISCIII on Research and Animal Welfare (CEIyBA number CBAO6_2018; -PROEX 161_18) under the provisions of RD53/2013 law. Any animal showing evident signs of distress (piloerection, lethargy, drop in temperature) was excluded from the manipulation to provide means for recovery. If severe distress persisted or if an animal lost more than 20% of its weight in three days, it was euthanized to avoid further suffering. The total number of animals for each experimental procedure is indicated in the figure legends. Animals were anesthetized with 4–5% isoflurane, followed by a cardiac puncture to collect the maximum volume of blood.

We employed randomization to allocate the 28 mice into three groups (ND, HFD-Mthistle, and HFD-control) to minimize selection bias and to ensure that the groups were comparable. Specifically, we used a computer-generated randomization sequence to assign each mouse to a group. This process was conducted by an independent researcher who was not involved in the subsequent experimental procedures, ensuring allocation concealment. Additionally, we implemented blinding procedures to reduce bias during data collection and analysis. The researchers conducting the experiments and analyzing the data were blinded to the group assignments. This was achieved by coding the groups with identifiers that did not reveal their allocation, and the codes were only revealed after the data analysis was completed.

### 2.3. Glucose Tolerance Test (GTT), Insulin Tolerance Test (ITT), and the Index of Homeostasis Model Assessment of Insulin Resistance (HOMA/IR)

For the glucose tolerance test (GTT), mice were fasted for a period of 16 h, and 2 g/kg of glucose (0.5 g/mL) was injected intraperitoneally. Then, blood glucose was measured at different time points (0, 15, 30, 60, 120, and 180 min) using a portable glucometer (Menarini Diagnostics, Florence, Italy). Insulin was also determined at time 0, prior to the glucose injection, as previously described [13].

For the insulin tolerance test (ITT), insulin was injected intraperitoneally (0.75 U/kg) (Insulin Humulina 100 U/mL, Lilly, Indianapolis, IN, USA) [14]. Then, blood glucose levels were determined at 0, 15, 30, 60, 120, and 180 min.

The index of Homeostasis Model Assessment of Insulin Resistance (HOMA/IR) was calculated applying the validated equation HOMA/IR = Fasting Glucose × Fasting Insulin/22.5 as previously described [13].

Plasmas were obtained by 10 min of centrifugation at 12,000 rcf in a centrifuge at 4 °C. The concentration of fasting insulin was then determined using the Elisa kit obtained from Crystal Chem (Inc-90080; Elk Grove Village, IL, USA).

### 2.4. Indirect Calorimetry

Indirect calorimetry analysis was conducted using metabolic cages (Oxylet Panlab Harvard Apparatus, Holliston, MA, USA) as previously described [15].

For the stabilization of oxygen and carbon dioxide fluxes, the weight of solid tissues obtained by dual X-ray absorption densitometry (GE Medical Systems PIXImus Lunar Densitometer, Chicago, IL, USA) was registered for a period of 72 h. Images were taken every 5 min and converted into masses of solid or fat tissues. Then, the total volume of oxygen (VO_2_) consumed, the total volume of carbon dioxide (VCO_2_) expelled, the activity of the mice, and the intake of food and water were monitored for another 72 h, in cycles of 12 h of light and 12 h of darkness.

For the calculation of EE, we applied the modified Weir equation, which is based on the volume of oxygen (VO_2_) consumed and the volume of carbon dioxide (VCO_2_) produced. This equation relies on average energy conversion factors of 3.941 for O_2_ and 1.106 for CO_2_ in mice [16]. Due to logistical constraints, only 8 animals per experimental group could be analyzed, as the metabolic chambers can accommodate only 8 animals per chamber.

All the analysis was performed separately for the repeated measurements taken on the third day, during the day or the night, due to the different physiological behavior expected in these two periods.

### 2.5. Cold Chambers

Insulated cages were placed in a cold chamber and the temperature of each mouse was monitored using a rectal probe at time 0, after a period of 72 h of acclimatation at 18 °C, and after 24 h of cold stress exposure at 4 °C [15]. At the end of the experiment, WATs and BATs were collected for subsequent molecular analysis.

One mouse of the HFD-control showed signs of stress, such as piloerection and tremors; therefore, we decided to remove it from this study. Thus, the cold stress chamber experiment was conducted with 7 HFD-control mice and 8 HFD-Mthistle mice.

### 2.6. Gene Expression Analysis

RNA was extracted from the different collected tissues. To avoid RNA degradation, the processing of all tissues was carried out on dry ice. An amount of 1–1.5 mL of phenol (TriReagent, Sigma, St. Louis, MO, USA) was added, and the tissue was homogenized with a T10 IKA homogenizer. Total RNA was quantified (UV-Vis spectrophotometer NanoDrop 2000/2000c, ThermoFisher Scientific, Waltham, MA, USA) and kept at −80 °C for future analysis.

A total of 1 μg of RNA was retrotranscribed into cDNA (high-capacity RNA-to-cDNA Master Mix kit, Life Technologies-ThermoFisher) in a Thermal Cycler equipment (Applied Biosciences 2720, Waltham, MA, USA), as previously described [15].

Quantitative real-time PCRs (qRT-PCRs) were carried out on a 7900HT Real-Time PCR apparatus using Taqman Gene Expression Master Mix and Taqman-specific probes (Supplementary Table S1). Analysis of the differential gene expression was carried out using Expression Suite Software version 1.0.4 and applying the 2^−ΔΔCt^ method Beta-2-Microglobulin (B2M) and/or 18S RNA was used as housekeeping genes.

### 2.7. Statistical Analysis

Data were collected for as many samples/mice as possible, although technical limitations reduced the number of replicates in some assays. To determine statistical differences among groups for the analysis of GTT, ITT, and gene expression, one-way analysis of variance (ANOVA) followed by Bonferroni’s post-test was used. All statistically significant values are indicated with asterisks based on their *p*-value. * *p* < 0.05, ** *p* < 0.01, *** *p* < 0.001. All graphical representations show the results as the mean ± standard deviation of the averages (SEM) of the experiments.

The statistical program R version 3.6.1 was used to conduct a comparative analysis of the indirect calorimetries. Both the average values and the area under the curve (AUC), obtained through trapezoidal integration on the third day, were modeled. Linear mixed models were employed to appropriately account for intra-mouse correlation, using a categorical variable as a random factor. Fixed factors included the weight of solid tissues (previously obtained by densitometry), time 0 h as the baseline, and an interaction between a three-level factor corresponding to the day of analysis (day 1, 2, or 3) and a two-level factor corresponding to the treatment (extract versus control). Additionally, marginal tests were performed to assess the significance of the treatment.

Excel Professional Plus 2016 and GraphPad Prism 8.0.1 software were used for graphical representations.

## 3. Results

Previously, we described that Mthistle augmented the expression of thermogenic markers in an in vitro model of differentiated human adipocytes (SGBS). These effects were corroborated by means of functional experiments where Mthistle reduced the total neutral lipid content and augmented the FAO and oxidative respiratory capacity of differentiated human adipocytes [12]. Therefore, in this study, we aimed to evaluate the in vivo potential of Mthistle to alleviate the metabolic stress associated with high-fat diet (HFD)-induced obesity (DIO) by means of the activation of thermogenesis.

### 3.1. Study Workflow

A total of 28 C57BL/6HsdOla male mice of 6 weeks of age were randomly allocated into three different groups: Group 1—normal-diet control group (ND-control) (N = 6); Group 2—high-fat-diet control group (HFD-control) (N = 10); and Group 3—high-fat-diet Mthistle group (HFD-Mthistle) (N = 12). The randomization sequence was generated using a random number table to ensure unbiased allocation. To minimize potential confounders, we standardized the experimental conditions by conducting all treatments and measurements at the same time of day.

**Figure 1:**
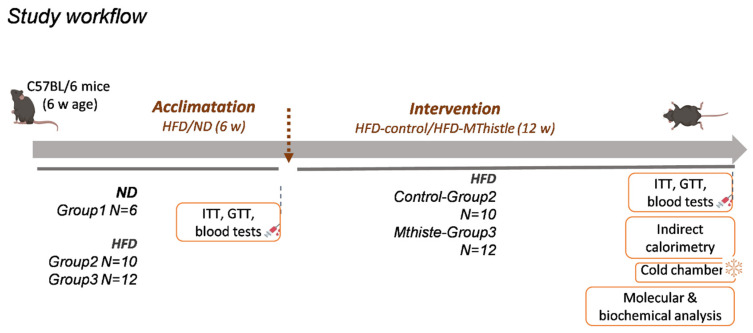
Study workflow and main readouts of this study (insulin and glucose tolerance tests, indirect calorimetry, cold stress exposition, and molecular analysis).

For the first six weeks prior to the intervention, mice in groups 2 and 3 were fed with a high-fat diet (HFD) to induce metabolic disturbances associated with weight gain. Following this period, the administration of Mthistle or vehicle began, continuing alongside the HFD. Mthistle was dissolved in drinking water and administered via gastric gavage (0.8 g of the extract per kg of mice) during three days per week for 12–14 weeks. Figure 1 shows the study workflow and main readouts of this study (insulin and glucose tolerance tests, indirect calorimetry, cold stress exposition, and molecular determinations).

### 3.2. HFD-Mthistle Showed an Improvement on Insulin Sensitivity Compared to HFD-Control

**Figure 2:**
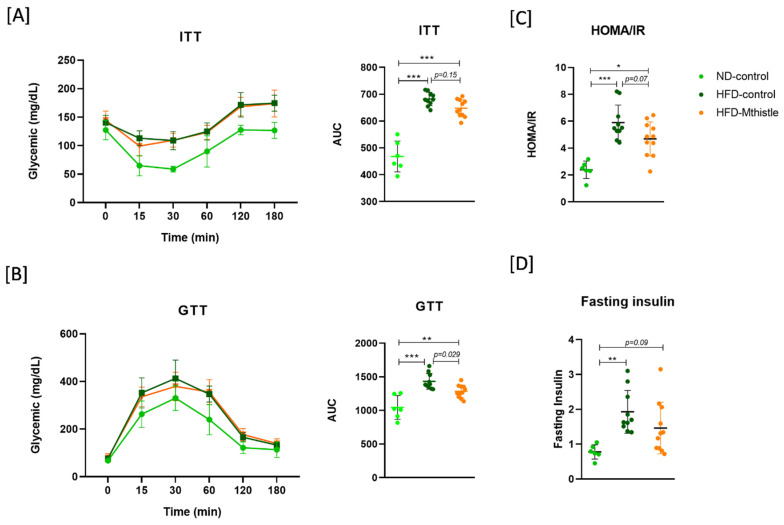
Effect of Mthistle treatment on glycemia. Graphical representation of the average glucose levels for each experimental group, HFD-control, HFD-Mthistle, and ND, at different times (**A**) for insulin tolerance test (ITT) and the corresponding area under the curve (AUC), and (**B**) for the glucose tolerance test (GTT) and the corresponding AUC. (**C**) Insulin resistance index (HOMA-IR) and (**D**) fasting insulin levels. Graphs represent the mean ± standard deviation of 6–12 animals per experimental group (ND: N = 6; HFD-Control: N = 10; HFD-Mthistle: N = 11). * *p* < 0.05; ** *p* < 0.01; *** *p* < 0.001.

After confirming the pre-diabetic situation in the HFD group, where plasma glucose levels and the HOMA-IR index were higher compared to the ND group (Supplementary Figure S1), the intervention with Mthistle (HFD-Mthistle, N = 11) or vehicle (HFD-control, N = 10) in combination with HFD was initiated for 12 weeks. The normal-diet group (ND group, N = 6) was maintained for comparison purposes. No significant differences were found in the maximum peaks of the glycemic curves of GTT or ITT between HFD-Mthistle and HFD-control. However, there was a tendency towards a reduction in AUC of ITT (Figure 2A) and a significant reduction in AUC of GTT (*p*-value 0.029) of HFD-Mthistle compared to HFD-control (Figure 2B). Importantly, fasting insulin levels were statistically significantly lower in HFD-Mthistle compared to HFD-control, while no significant differences were found between HFD-Mthistle and ND (*p*-value 0.09) (Figure 2C). In addition, the calculated HOMA-IR index of HFD-Mthistle showed a tendency towards reduction compared to HFD-control (*p*-value 0.07) (Figure 2D), although it did not return to the levels of ND (Figure 2).

### 3.3. HFD-Mthistle Showed a Decrease in Food Intake and Weight Compared to HFD-Control

**Figure 3:**
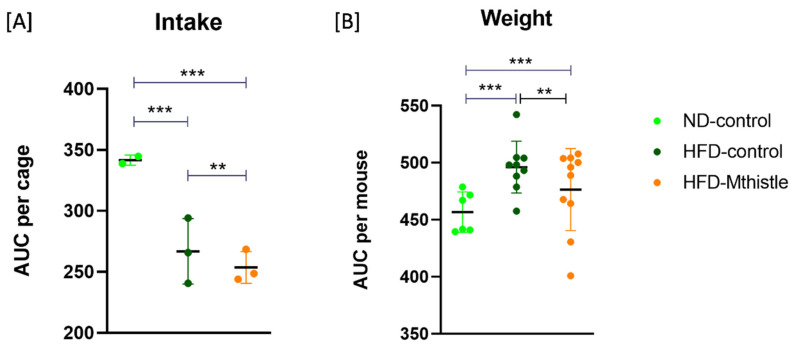
Intake and weight variation of each study group. (**A**) Area under the curve (AUC) of food intake per cage, and (**B**) weight variation per mouse in ND (N = 6), HFD-control (N = 9), and HFD-Mthistle (N = 10). Graphs represent the mean ± standard deviation per experimental group. ** *p* < 0.01; *** *p* < 0.001.

The weight and food intake of animals were monitored weekly (Supplementary Figure S2). A significant increase in food intake was observed in ND compared to HFD-control and HFD-Mthistle, due to the satiating effect of the HFD. However, HFD-Mthistle showed a significant decrease in food intake compared to HFD-control (Figure 3A). In addition, although both HFD-control and HFD-Mthistle significantly increased their weight compared to ND, HFD-Mthistle showed a statistically significant decrease in weight compared to HFD-control (Figure 2B). We also monitored the weight of different types of adipose tissue (AT): brown adipose tissue (BAT), epididymal white adipose tissue (eWAT), and inguinal white adipose tissue (i-WAT). Importantly, the weight of eWAT in HFD-Mthistle was similar to that of ND (*p* = 0.167), while the eWAT of HFD-control was significantly increased compared to ND and HFD-Mthistle (Supplementary Figure S3). This result suggests that Mthistle may help reduce the pro-inflammatory eWAT associated with HFD.

### 3.4. HFD-Mthistle Showed an Increase in Energy Expenditure Compared to HFD-Control

**Figure 4:**
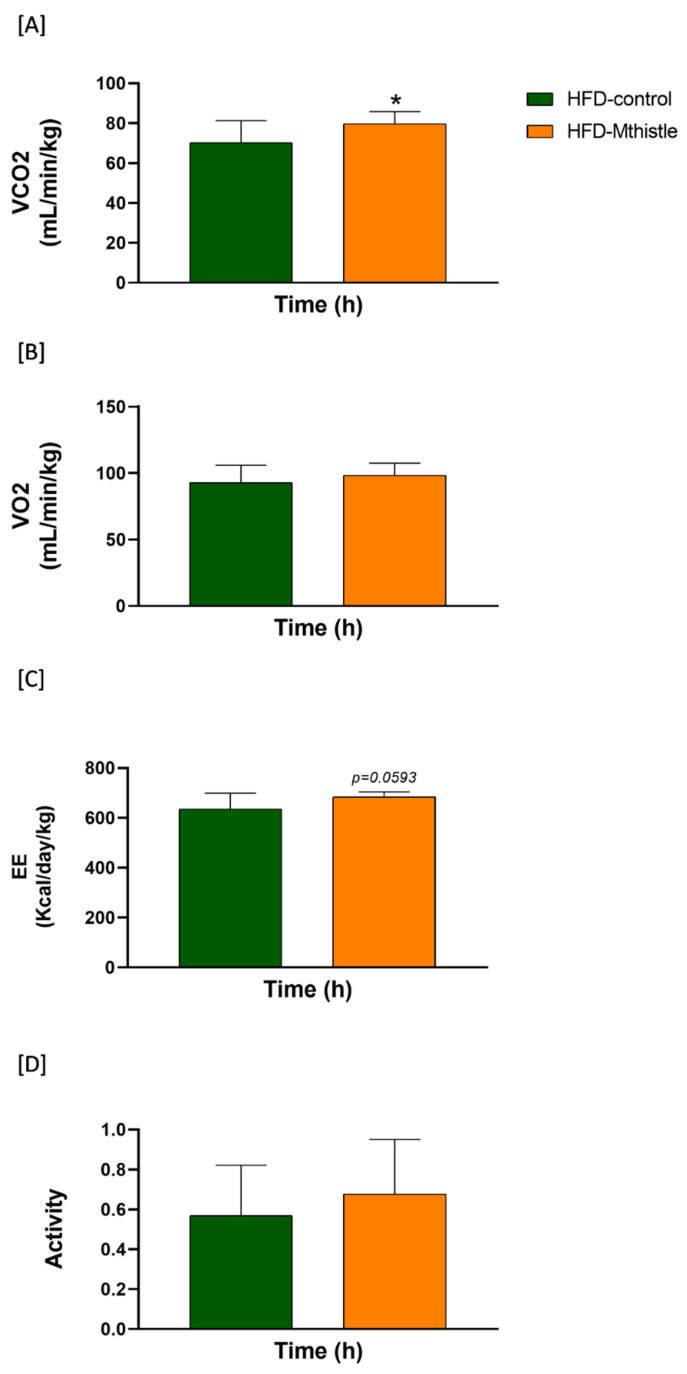
Graphical representation of the variables measured by indirect calorimetry in metabolic chambers for HFD-control and HFD-Mthistle. Values recorded on the third day for the volume of oxygen (VO_2_) (**A**), volume of carbon dioxide (VCO_2_) (**B**), energy expenditure (EE) (**C**), and activity (**D**) are represented. Graphs represent the mean ± standard deviation of 8 mice per experimental group, as the metabolic chambers can accommodate only 8 animals per chamber. * *p* < 0.05.

To delve into the underlying mechanisms involved in the improved insulin sensitivity observed in HFD-Mthistle compared to HFD-control and considering that Mthistle extract reduced the eWAT weight compared to HFD-control, we applied indirect calorimetry to quantify parameters associated with systemic energy expenditure (EE). As shown in Figure 4, a significant increase in VCO_2_ (*p*-value = 0.033), an indirect measurement of metabolic expenditure, and a tendency to increase EE (*p*-value = 0.059) were observed in HFD-Mthistle (N = 8) compared to HFD-control (N = 8). Since we did not observe differences in the activity of mice, these results suggest an increase in lipolysis and/or WAT browning in HFD-Mthistle compared to HFD-control [17].

### 3.5. HFD-Mthistle Preserved Body Temperature After Cold Exposure Compared to HFD-Control

As previous results suggest that Mthistle may promote an increase in systemic energy expenditure, possibly by augmenting the β-oxidation of FAs, we next evaluated the effect on body temperature after cold exposure, as a more direct measurement of thermogenic activation.

**Figure 5:**
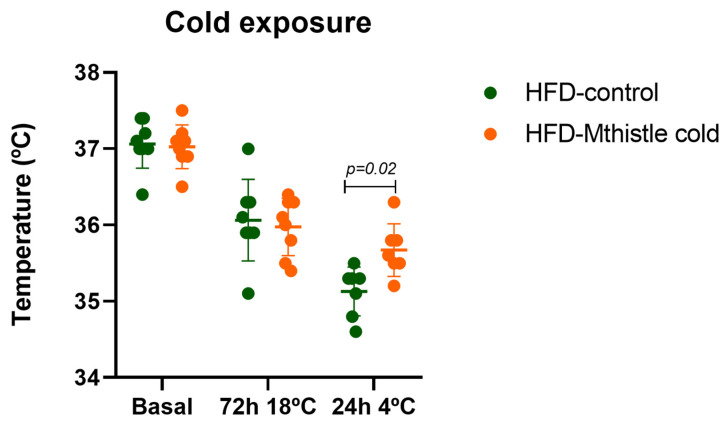
Rectal temperature measurements of mice exposed to cold in HFD-Mthistle (N = 8) and HFD-control (N = 7). Graphs represent the mean ± standard deviation per experimental group.

For this purpose, after an acclimatation period of 72 h at 18 °C, animals were maintained at 4 °C for 24 h. As shown in Figure 5, Mthistle helped to preserve body temperature compared to the HFD-control group. It is important to note that in both groups, body temperature was drastically reduced within the first hours of cold exposure, making it very difficult to reverse this thermal change in such a short period (24 h of extreme cold) with Mthistle treatment alone. However, HFD-Mthistle showed a significant improvement in temperature compared to HFD-control (*p*-value 0.02).

### 3.6. Mthistle Modulated the Expression of Key Metabolic Genes Involved in Browning and Mitochondrial Function in eWAT, iWAT, and BAT

To investigate the underlying mechanisms by which Mthistle protects against metabolic stress induced by a high-fat diet (HFD), various types of fat tissues—epididymal white adipose tissue (eWAT), inguinal white adipose tissue (iWAT), and brown adipose tissue (BAT)—were collected. These tissues were examined for the expression of genes related to thermogenesis, mitochondrial uncoupling, mitochondrial oxidative capacity, mitochondrial biogenesis, oxidative stress, and fatty acid β-oxidation, among others.

**Figure 6:**
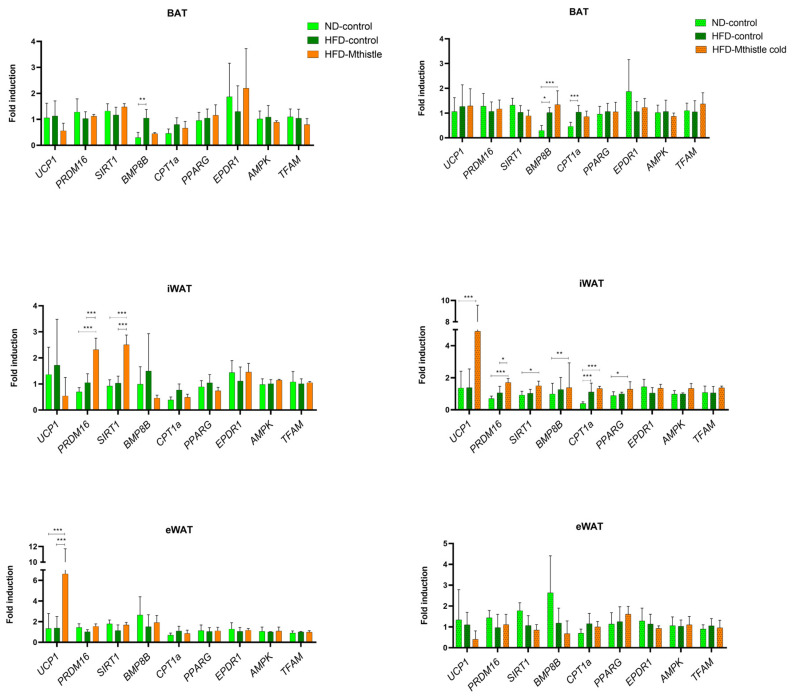
Gene expression levels of targets related to thermogenesis were measured in different types of adipose tissues—brown (BAT), inguinal white (iWAT), and epididymal white (eWAT)—across ND (N = 4), HFD control (N = 6), and HFD-Mthistle (N = 6) with and without exposure to cold stress. Graphs represent the average of the normalized expression levels relative to the ND group ± SEM per experimental group. * *p* < 0.05; ** *p* < 0.01; *** *p* < 0.001.

First, the expression of the *UCP1* gene, which induces mitochondrial uncoupling in adaptive thermogenesis, was analyzed. As shown in Figure 6, *UCP1* gene expression was upregulated in the iWAT of HFD-Mthistle, after cold exposition, compared to HFD-control. In addition, *UCP1* was also significantly upregulated in the eWAT of HFD-Mthistle compared to HFD-control. However, this effect was reversed after exposure to low temperatures, possibly because this stress may also promote *UCP1* expression in the eWAT to maintain body temperature [18,19].

*PRDM16* gene expression was significantly upregulated in the iWAT of HFD-Mthistle compared to ND and HFD-control, both under normal and cold-exposed conditions.

*SIRT1* was also upregulated in the iWAT of HFD-Mthistle compared to HFD-control, although the effect was more pronounced in the absence of cold exposure. This may be because *SIRT1* expression could also be induced in HFD-control under low-temperature stress, diminishing the significance of the effect [20,21,22,23,24].

We also observed the upregulation of *CPT1A* gene expression in the iWAT of HFD-Mthistle, a gene involved in mitochondrial fatty acid β-oxidation, in line with the increase in the VCO_2_ detected by indirect calorimetry [25,26] (Figure 4).

Importantly, *BMP8B* gene expression, which is a well-known transcription factor implicated in the activation of the browning process [27], was significantly upregulated in the BAT and iWAT tissues of HFD-Mthistle compared to HFD-control under low-temperature stress. Additionally, *PPARγ*, a master regulator of the thermogenic program in brown and beige adipocytes, was specifically upregulated in the iWAT of the HFD-Mthistle group under low-temperature exposure.

Overall, the main metabolic effects related to the activation of thermogenesis were observed in the iWAT, which is the WAT most prone to be reprogrammed to a beige phenotype.

### 3.7. Mthistle Diminished the Expression of Inflammatory Markers in AT

**Figure 7:**
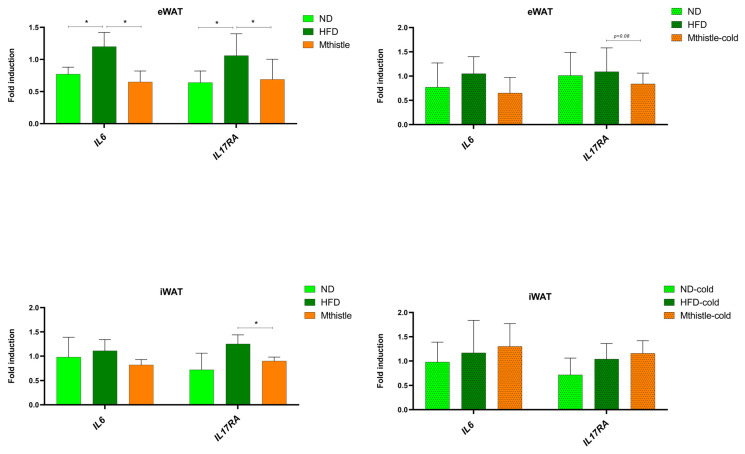
Gene expression levels of inflammatory biomarkers in inguinal white (iWAT) and epididymal white (eWAT) adipose tissues. Gene expression of *IL6* and interleukin 17A receptor (*IL17RA*) in eWAT and in iWAT of HFD-control, HFD-Mthistle, and ND, under normal conditions or after exposure to cold stress (72 h at 18 °C followed by 24 h at 4 °C). Graphs represent the average of the normalized expression levels relative to ND ± SEM (ND, N = 4; HFD-Mthistle, N = 6; HFD-control, N = 6). * *p* < 0.05.

As meta-inflammation is highly associated with WAT atrophy and immune system dysfunction during obesity and aging [28], we next analyzed the effect of Mthistle on inflammation markers in WAT. As shown in Figure 7, *IL6* gene expression levels were reduced in HFD-Mthistle compared to HFD-control, specifically in the eWAT, which correlates with visceral WAT in human obesity. In addition, gene expression levels of the interleukin 17A receptor (*IL17RA*), which has been extensively linked to inflammatory obesity [29,30], was also significantly reduced in the eWAT of HFD-Mthistle compared to HFD-control. These effects were lost under cold exposure, suggesting that this thermal stress may also induce thermogenesis in HFD-control. No effects were observed in the iWAT of the HFD-Mthistle group compared to the HFD-control group.

## 4. Discussion

A sedentary lifestyle and overfeeding have been demonstrated to disrupt the metabolic function of specific tissues such as adipose tissues (ATs), muscles, the pancreas, and the liver. Obesity is characterized by the progressive loss of brown adipose tissue (BAT), along with the reduction in subcutaneous white adipose tissue (sWAT) and the increase in visceral white adipose tissue (vWAT), which promotes a senescent-associated secretory phenotype (SASP) and local and systemic inflammation. Moreover, adipose tissue resident immune cells are turned into pro-inflammatory type 1 immune cells, exacerbating meta-inflammation. Although the anti-inflammatory capacity of thermogenic adipose tissues is widely recognized, the key mediators involved are still poorly characterized. Similar to obesity, aging is characterized by a low grade of chronic inflammation associated with the progressive loss of thermogenic ATs [31]. Inflammaging is also the main cause of age-related diseases such as type 2 diabetes and immunometabolic injuries, mainly infections and cancer. On the contrary, healthy aging is accompanied by well-functioning adipose tissue in terms of insulin sensitivity and anti-inflammatory profile [32]. Thus, it has been proposed that preserving functional thermogenic adipose tissue could represent an effective strategy to reduce meta-inflammation during obesity and to prolong health span [3].

Precision nutrition has emerged as a key discipline that integrates data from omics technologies and traditional nutritional assessments to provide personalized dietary intervention strategies against chronic diseases and aging. It considers several aspects, such as the different responses of individuals to dietary exposure, influenced by the gut microbiome and individual genetic susceptibilities, and the nutritional situation of the individuals, highly influenced by lifestyle factors. Moreover, precision nutrition also aims to identify biomarkers to predict disease risk as well as molecular targets susceptible of being modulated by personalized interventions [33]. Thus, diet-based interventions, including bioactive compounds from natural sources as nutraceutics, provide complementary tools to keep a protective thermogenic and anti-inflammatory profile to restore metabolic homeostasis during obesity and aging [33,34].

In a previous work, Mthisle extract, 50% rich in silymarin, induced thermogenesis in SGBS adipocytes, leading to a reduction in the total neutral lipid content by increasing fatty acid β-oxidation [10]. Thus, herein, we applied a preclinical model of HFD-induced obesity as a model of metabolic inflammation to evaluate the potential of Mthistle to relieve metabolic stress and inflammation by means of the activation of the thermogenic program in vivo.

First, an anorexigenic effect was observed in HFD-Mthistle compared to HFD-control, concomitant with a statistically significant reduction in body weight and inflammatory eWAT weight. These effects seemed to be on the bases of the improved systemic glucose homeostasis and insulin sensitivity observed in HFD-Mthistle compared to HFD-control [35,36,37]. In line with this, several studies have also described the effect of pure silymarin reducing the WAT weight after HFD in preclinical models [36,38,39] and in human nutritional intervention trials [40,41]. Soto C. et al. also demonstrated that silymarin favors the recovery of the morphology and endocrine function of damaged pancreatic tissue in diabetic rats [38]. Nevertheless, none of these studies have evaluated the role of Mthistle in the activation of functional thermogenesis and the “beigeing” phenotype of WAT to augment insulin sensitivity.

Herein, Mthistle increased the systemic energy expenditure compared to the HFD-control, suggesting an increase in lipolysis and WAT browning [17]. In addition, the reduction in eWAT weight was reduced in HFD-Mthistle compared to the HFD-control. These results are also in line with the improved preservation of body temperature after cold exposure in HFD-Mthistle compared to HFD-control.

The analysis of gene expression on the different types of adipose tissues—epidydimal white adipose tissue (eWAT), inguinal adipose tissue (iWAT), and brown adipose tissue (BAT)—suggests a relevant role of Mthistle in activating the browning program mainly in the iWAT, which is the adipose tissue more susceptible to be reprogrammed to a beige thermogenic phenotype. Thus, *UCP1* gene expression, a key promoter of mitochondrial uncoupling that takes place in adaptive thermogenesis, was significantly induced in the eWAT of HFD-Mthistle compared to HFD-control [18,19], and in the iWAT of HFD-Mthistle after cold exposition, which may be related to the higher body temperature preservation in HFD-Mithistle compared to HFD-control. In addition, *PRDM16* and *SIRT1* genes, implicated in the activation of browning and mitochondrial oxidative capacity, were significantly upregulated in the iWAT of HFD-Mthistle compared to the HFD-control group, both in normal and after cold exposure [20,21,22,23,24]. It is noteworthy that SIRT1 upregulation has been extensively described as a promoter of healthy aging by means of its effects on improving insulin sensitivity and the promotion of the polarization of macrophages towards an anti-inflammatory phenotype [42]. SIRT1 is also implicated in the promotion of healthy adipogenesis, without visceral fat induction [43]. On the contrary, mice lacking the *SIRT1* gene develop exacerbated hepatic steatosis, increased insulin resistance (IR), ectopic lipid accumulation, and AT inflammation, after HFD-induced obesity, worsening and accelerating the aging process [44]. A significant upregulation of *CPT1A*, a gene involved in mitochondrial fatty acid β-oxidation [25,26], was also found, specifically in the iWAT of the HFD-Mhistle group compared to the HFD-control group, which is in line with the increase in the VC0_2_ detected by indirect calorimetry. In addition, key transcription factors implicated in the activation of the thermogenic program in brown and beige adipocytes, such as *BMP8B* [27], and *PPARγ* were significantly upregulated in the iWAT of the HFD-Mthistle group exposed to low temperature compared to the HFD-control group. Interestingly, the antidiabetic thiazolidinediones (TZDs, e.g., rosiglitazone and pioglitazone), which are agonists of PPARγ, have been shown to exert their metabolic benefits by means of the PPARγ-mediated anti-inflammatory effects [45,46].

In addition, MThistle reduced *IL6* gene expression in the eWAT, which correlates with visceral WAT in unhealthy obesity. This effect was lost under cold exposition, suggesting that thermic stress may also upregulate thermogenesis in HFD-control, reducing inflammation. No effects were observed on iWAT in HFD-Mthistle compared to HFD-control, suggesting an improvement in the pro-inflammatory profile associated with this tissue. Importantly, the gene expression of the interleukin 17A receptor (*IL17RA*, interleukin 17 receptor A) extensively linked to inflammatory obesity [29,30] was significantly reduced in the eWAT of HFD-Mthistle compared to HFD-control. This is important as inflammatory immune cells compete with adipocytes for nutrients. Type 1 inflammatory immune cells depend mainly on glycolysis, while pro-resolving and anti-inflammatory type 2 macrophages and Treg cells rely on mitochondrial FAO. The reduction in circulating glucose levels may contribute to balance the inflammatory/anti-inflammatory homeostasis of immune cells. These results are in line with other studies where silymarin-rich extracts showed similar results in terms of the reduction in inflammatory interleukins versus HFD controls [47]. Nevertheless, a direct link between the thermogenic effect and the inflammatory status of e-WAT was not described in these studies [48].

## 5. Conclusions

In summary, it can be concluded that Mthistle significantly increases markers of thermogenesis and the browning of inflammatory WAT (e.g., increased energy expenditure and preservation of body temperature after cold exposition) and reduces AT inflammation to restore metabolic homeostasis (e.g., insulin sensitivity) and balance type 1/type 2 immune function. Thus, Mthistle may be proposed as a potential nutraceutic in the treatment of obesity and accelerated aging by means of its effects on the induction of the thermogenic program to relieve metabolic inflammation.

### Future Directions

One of the weaknesses of this study is that it has been conducted only in one preclinical model of obesity. We decided to evaluate the effect of Mthistle in a preclinical model of obesity induced to mimic environmental and dietary factors contributing to obesity in humans. The C57BL/6 strain, when exposed to DIO, displays increased glucose intolerance, and moderate insulin resistance. Nevertheless, confirming the efficacy of Mthistle in a different model of obesity such as ob/ob mice will enhance and expand the robustness of our conclusions.

In addition, because our study was conducted only in a preclinical model, it remains to be determined whether our findings can directly translate to humans. In adult humans, BAT is present in multiple locations, and UCP1-positive adipocytes in the supraclavicular region show a molecular signature similar to that of mouse beige adipocytes, whereas the deep neck region contains classical brown adipocyte-like cells.

It is generally accepted that BAT activation and/or WAT browning have various health benefits. This idea is specifically applicable to individuals consuming excessive nutrients, particularly those with obesity or metabolic syndrome, but not to all other people. Within the framework of precision nutrition, it is crucial to stratify individuals based on their nutritional and metabolic conditions to avoid deleterious effects in vulnerable populations such as cachexic cancer patients and individuals with infectious diseases. Moreover, it should be mandatory to develop innovative strategies to tightly regulate BAT activation and/or WAT browning using a brake-switch system. Recently, *SIRT7* has been demonstrated to allow the switch-off in energy consumption in pathological conditions such as cachexia to avoid excessive BAT activation and/or WAT browning [49].

## Data Availability

Study data are available in the Supplementary Materials section and can be accessed upon request from the corresponding author.

## Supplementary Materials

The following supporting information can be downloaded at https://www.mdpi.com/article/10.3390/nu16234166/s1. Figure S1: Weight of the distinct types of adipose tissues—epididymal white adipose tissues (eWAT), inguinal white adipose tissue (iWAT), and brown adipose tissue (BAT)—of HFD-Mthistle, HFD-control, and ND. Figure S2: Insulin and glucose tolerance tests of mice under an HFD for 6 weeks compared to ND. The area under the curve (AUC) was calculated for each experiment. Asterisks indicate statistically significant differences between HFD and ND. *, *p* < 0.05. Figure S3: Monitoring of the food intake, drinking water, and activity of mice in the metabolic chambers in HFD-Mthistle and HFD groups (N = 8). Table S1: List of Taqman probes used in this study.
