# Supplementary figures and images for: Silymarin-Enriched Extract from Milk Thistle Activates Thermogenesis in a Preclinical Model of High-Fat-Diet-Induced Obesity to Relieve Systemic Meta-Inflammation
Source: Nutrients. 2024 Nov 30;16(23):4166. doi: 10.3390/nu16234166 (PMC11644670; doi:10.3390/nu16234166)

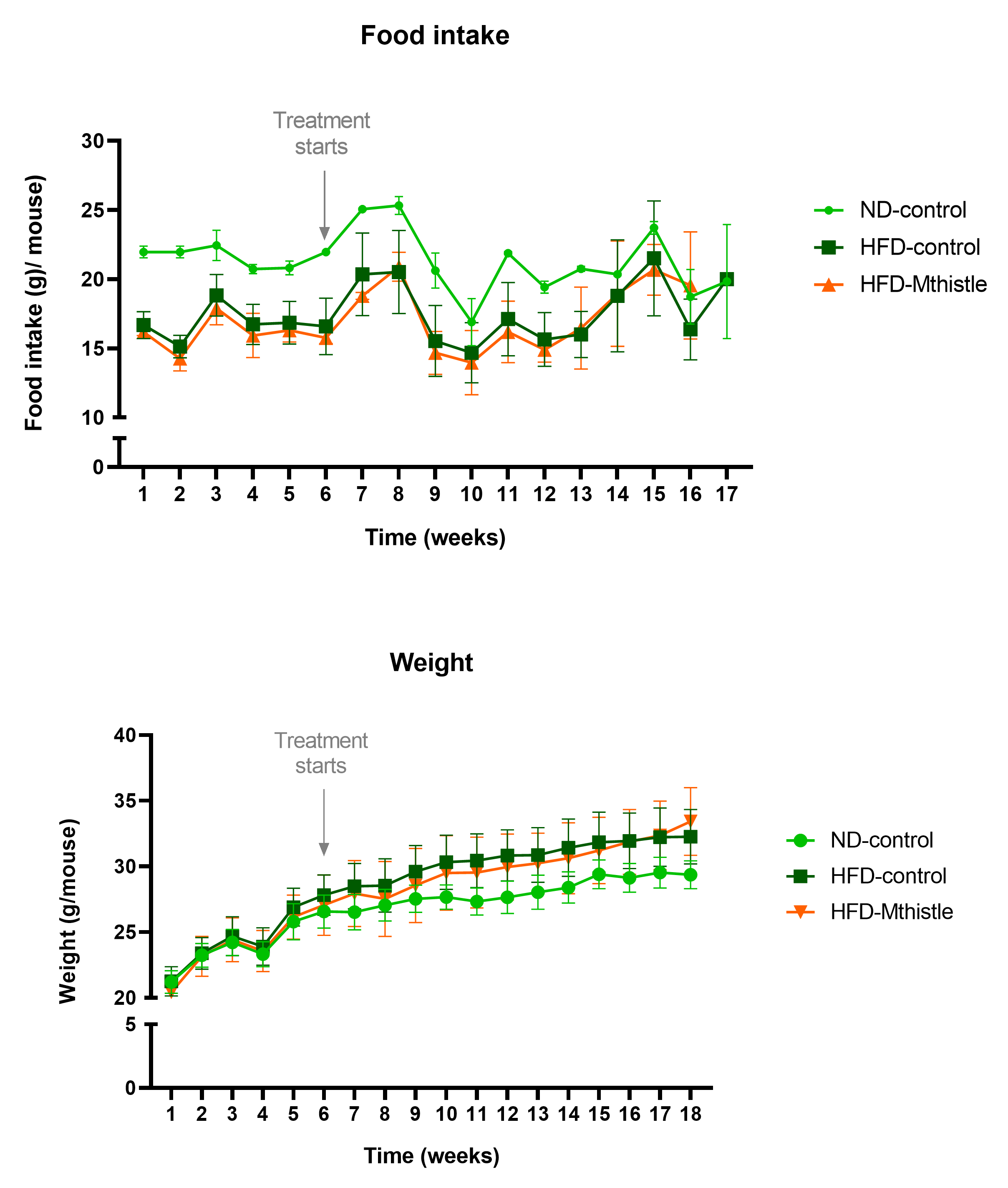

Supplement: Supplementary file 1 [file nutrients-16-04166-s001.zip › Supplementary Figure 2.png]

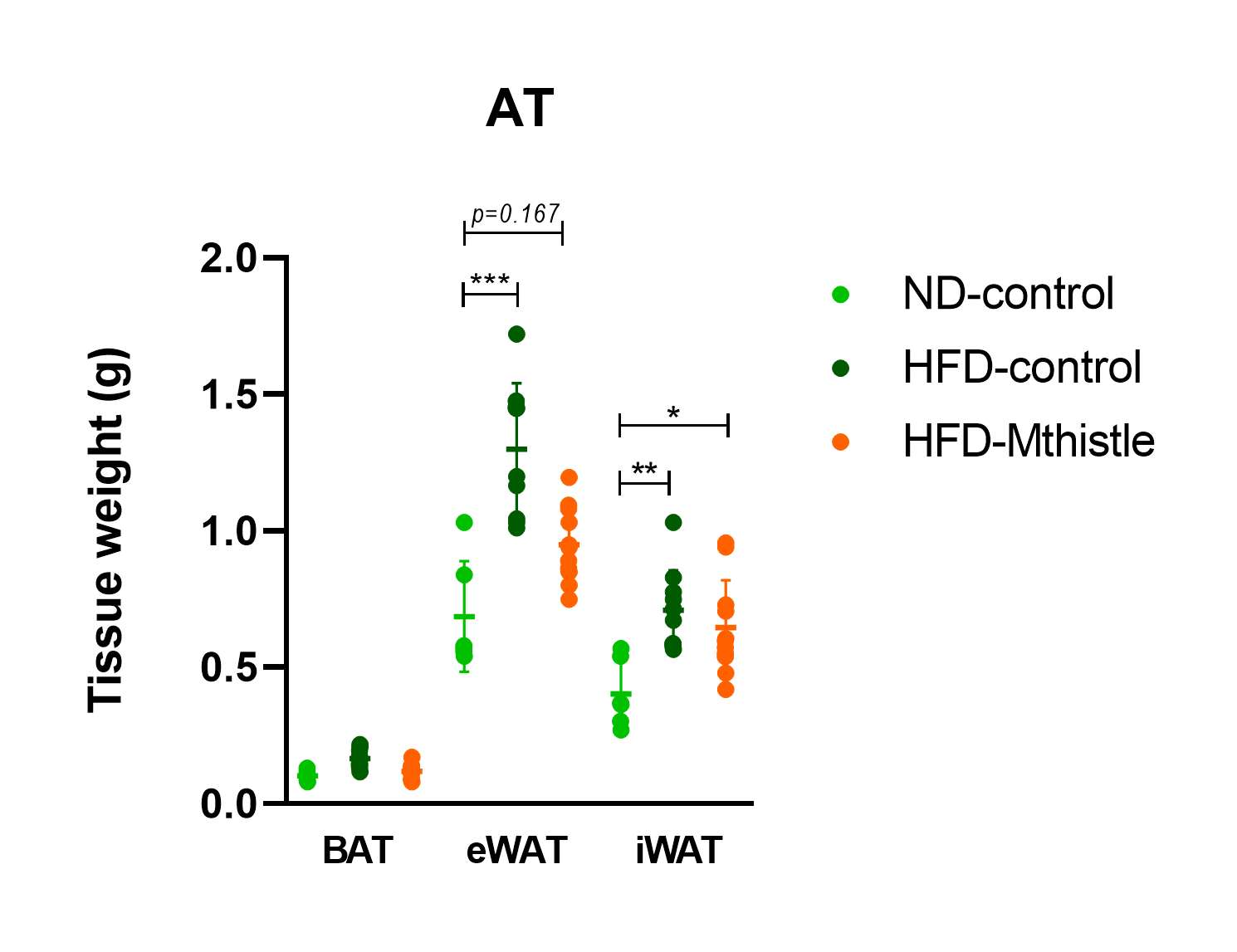

Supplement: Supplementary file 1 [file nutrients-16-04166-s001.zip › Supplementary Figure 3.png]

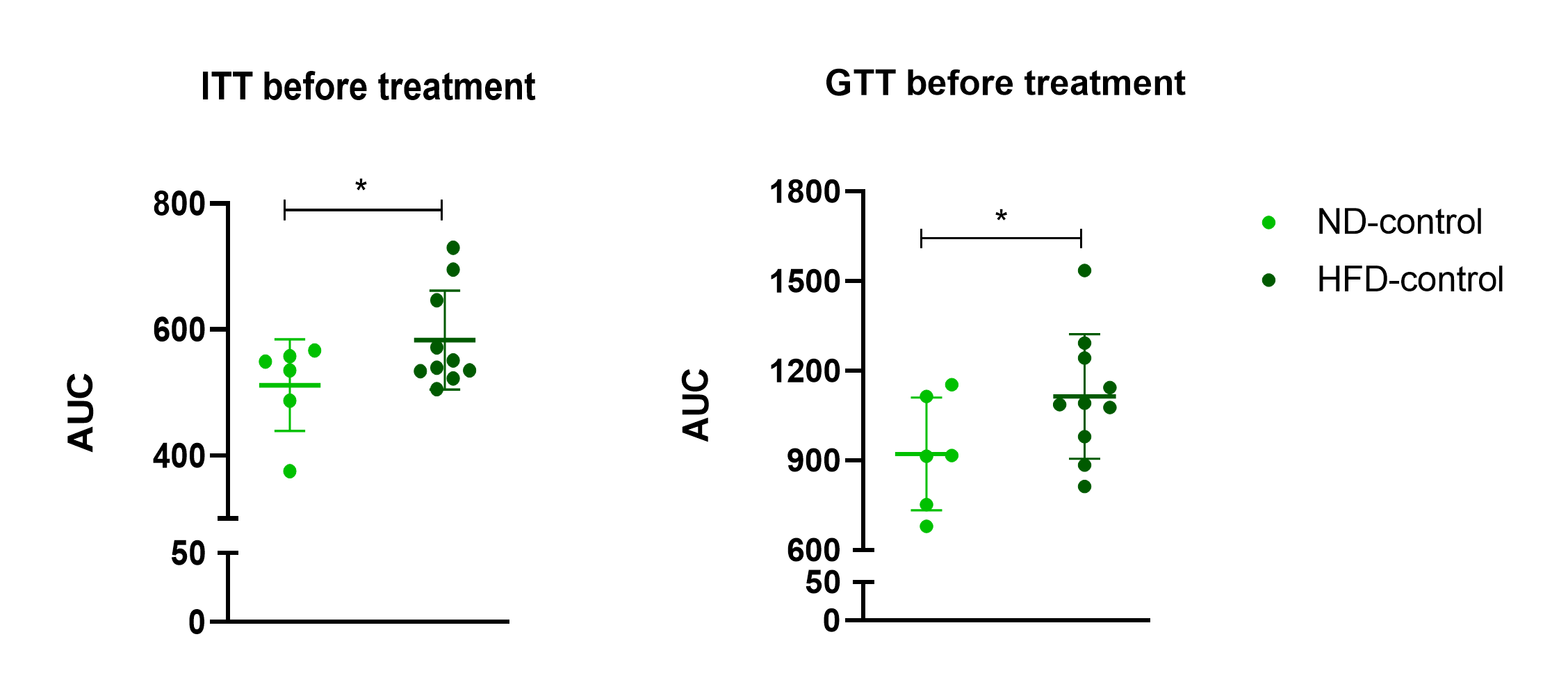

Supplement: Supplementary file 1 [file nutrients-16-04166-s001.zip › Supplementary Figure 1.png]
